# The Effect of Statin Therapy on Hemorheological Parameters of Patients with Clinically Silent Ischemic Foci of the Brain

**DOI:** 10.3390/ijms26157039

**Published:** 2025-07-22

**Authors:** Anna Marcinkowska-Gapińska, Izabela Siemieniak

**Affiliations:** 1 Department of Biophysics, Poznan University of Medical Sciences, 61-701 Poznan, Poland; 2 Rheological Laboratory, Department of Neurology, Poznan University of Medical Sciences, 61-701 Poznan, Poland

**Keywords:** hemorheology, blood viscosity, CSVCL, statin therapy, aggregability of erythrocytes, deformability of erythrocytes

## Abstract

Hemorheology is a branch of science that studies and explains the causes of blood flow disorders. In many vascular disorders whole blood viscosity, plasma viscosity, aggregability, and deformability of erythrocytes can be a diagnostic factor. In this paper we analyze whether statin therapy affects hemorheological values in a group of patients with clinically diagnosed silent ischemic foci of the brain (CSVCL). The study includes an analysis of the hemorheological parameter values such as whole blood viscosity, plasma viscosity, and selected biochemical parameters. Aggregability and deformability of erythrocytes were determined using the mathematical Quemada model. Our results indicate a modifying effect of statins on hemorheological parameters.

## 1. Introduction

Studies of hemorheological properties enable the explanation of blood supply disorders based on the determination of the values of physicochemical parameters of whole blood. Whole blood viscosity depends on the hematocrit value, plasma viscosity, and the erythrocyte tendency to aggregate and deform [1,2,3]. Plasma viscosity depends on its protein and lipid composition [4,5]. In the case of many vascular diseases, such as arterial hypertension, diabetes, and cardiovascular diseases, the analysis of rheological parameter values is still underestimated diagnostically [4,6]. Over the last 20 years, the results of several studies—conducted to determine whether changes in hemorheological parameters are important for the development of cerebral ischemia and whether they are a causative factor or only accompany other pathophysiological phenomena of the cerebral ischemia process [7,8,9]—have been published. Blood flow disorders are a condition for the occurrence of ischemic stroke in both the acute and chronic phase, transient cerebral ischemia, and the formation of so-called silent ischemic foci of the brain (CSVCL). Abnormalities in blood rheological parameters are observed in more than 40% of cases of people with cerebral ischemia [8,10,11,12]. Many researchers have found increased whole blood viscosity values in patients with cerebral ischemia compared to healthy controls [7,8,13]. Similarly, in chronic cerebral ischemia hemorheological changes are observed, such as increased blood and plasma viscosity, increased plasma fibrinogen concentration, increased hematocrit and red blood cell aggregability, and changes in erythrocyte deformability [6,7,8,10,13,14,15,16]. In patients with silent ischemic lesions, depending on the number of ischemic lesions in the brain detected in MRI—low-stage disease (0–3) and high-stage disease (>3)—it was shown that in the group of patients with high-stage disease, fibrinogen values were higher than in patients with low-stage disease but significantly lower compared to patients with chronic ischemic disease [13]. The incidence of clinically silent ischemic lesions in the brain increases with age, and the risk factors are consistent with the risk factors of cerebrovascular diseases, the most important of which, apart from age, are arterial hypertension, diabetes, atrial fibrillation, elevated cholesterol and homocysteine levels, the presence of atherosclerotic plaques in the carotid arteries, and tobacco smoking [17,18,19].

Epidemiological studies have shown that abnormal blood lipid levels are an important risk factor for atherosclerosis and cardiovascular diseases [20]. Increased triglyceride values increase blood and plasma viscosity, while high HDL values reduce plasma viscosity [15,20,21,22]. High lipid levels worsen the rheological properties of blood by increasing the formation of red blood cell aggregates and increasing plasma viscosity. Hemorheological disorders participate in the formation of atherosclerotic plaques in the walls of arteries. Hemorheological deviations such as increased plasma viscosity and erythrocyte aggregation and their poorer deformability are observed in various pathological conditions that promote the following atherogenesis: coronary heart disease, atherosclerosis, diabetes, obesity, hypertension, metabolic syndrome, or hypercholesterolemia [3,6,15,23]. Experimental models of mechanical properties of blood flow show characteristic blood flow disorders in the places of vessel branching, the so-called geometric parameters of atherosclerosis risk, predisposing to the formation of atherosclerotic plaques. The change in blood flow from laminar to turbulent promotes the formation of local secondary flows and causes local deterioration of hemorheological properties of blood. In the places of vessel bifurcation, the blood vessel wall is affected by variable shear stress (τ) [24,25].

Disturbances of the rheological properties of blood affect the slowing down of blood flow in vessels and, thus, the time of atherogenic particle retention at the vessel branching point. As a result of local action of variable shear stress (τ) due to mechanotransduction, an inflammatory response of the vessel wall, mechanical damage to the endothelium, and local conditions related to increased blood viscosity and increased aggregation of erythrocytes occur, leading to the formation of atherosclerotic plaques. The key role of fibrinogen in this phenomenon should be noted [26,27,28,29,30,31]. Fibrinogen is deposited in atherosclerotic plaques, transforming into fibrin and fibrinogen breakdown products, which have an atherogenic effect on the vessel wall, and erythrocytes increase platelet aggregation, contributing to the accumulation of both thrombocytes and leukocytes, giving rise to the development of a clot. The effect of erythrocytes on platelet activation is explained by increased prothrombotic activity of the erythrocyte cell membrane, increased endothelial adhesion, and decreased red blood cell deformability [26,27,28,29,30,31].

The lipid composition of the erythrocyte membrane is characterized by a state of dynamic equilibrium with plasma lipoproteins and reflects the processes occurring in the extracellular environment. In order to modify the development of atherosclerosis, drugs lowering the level of lipids in the blood serum are used [32,33,34]. High whole blood viscosity can also be reduced after statin therapy, and researchers suggest that drug administration could have a preventive effect in acute coronary syndrome [35]. Rasyid et al. [36] in their studies on ischemic strokes point out that the outcome of acute stroke is influenced by increased blood viscosity caused by increased levels of fibrinogen and LDL. In the conclusions of their work, the authors emphasize that fibrinogen, dyslipidemia, and hype viscosity are factors that should be controlled in patients with a stroke or at risk of stroke [36].

The aim of this work is to analyze the hemorheological data obtained during the measurement of the blood flow curve taken from people diagnosed with clinically silent ischemic foci of the brain in comparison to the control group—without visualized ischemic foci in the CNS in neuroimaging studies depending on statin therapy.

## 2. Results

Table 1 and Table 2 present the results of hemorheological and biochemical values for the group of patients and the control group not taking statins, and Table 3 and Table 4 present the results of hemorheological values for the group of patients and the control group taking statins. In the obtained results of hemorheological values for the people not taking statins (Table 1), statistical significance is observed in the group of patients compared to the control group in terms of the ability of erythrocytes to aggregate (the value of the Quemada model parameter k_0_ differed statistically at the level of *p* < 0.005) and the tendency of erythrocytes to deform (the level of significance for the Quemada model parameter k_∞_ *p* < 0.05). Based on the analysis of biochemical parameter values for people not taking statins (Table 2), statistically significant differences were observed for the group of patients compared to the control group in terms of IgM values (*p* < 0.0040, ESR (*p* < 0.049) and the albumin/globulin ratio (*p* < 0.009). Analysis of the values of hemorheological parameters obtained in the control group and the group of patients taking statins (Table 3) indicates statistically significant differences for the group of patients compared to the control group in terms of plasma viscosity η*p* (*p* < 0.03). Further differences in statistical significance were observed among the values of biochemical parameters obtained in the control group compared to the group of patients taking statins (Table 4) for the following parameters: IgM (*p* < 0.05), albumin/globulin ratio (*p* < 0.046), and cholesterol level (*p* < 0.04).

Comparison of hemorheological and biochemical parameter values in the group of patients divided into the group taking and not taking statins, with the statistical significance of differences indicated, is presented in Table 5 and Table 6. The observed differences were statistically significant in the case of plasma viscosity values (*p* < 0.007) and erythrocyte deformability (*p* < 0.01) (Table 5) and cholesterol level (*p* < 0.002) (Table 6).

## 3. Discussion

The mechanisms of blood flow disorders in cerebrovascular diseases, and, in particular, clinically silent ischemic changes in the brain, despite many studies conducted so far, still conceal many unknowns [17,18,37]. The formation of these changes is associated with circulatory disorders at the level of capillaries, where the condition for maintaining the flow is maintaining the balance of hemorheological parameters. Hemorheological disorders contributing to the formation of silent ischemic foci of the brain are associated with poorer deformability of red blood cells [17]. In the work of Marcinkowska-Gapińska et al., a correlation was found between the parameter of the Quemada model k_∞_ and plasma viscosity ηp. Furthermore, it was also observed that in the group of patients and the control group, the decrease in the ability of erythrocytes to deform coexist with the intensification of plasma viscosity [17]. These parameters are independent quantities, but they have an impact on the viscosity of whole blood. Plasma viscosity depends on its lipid and protein composition [4,5], and the deformability of erythrocytes is determined by a number of internal factors (erythrocyte internal viscosity) and external factors (surface area to volume ratio and erythrocyte membrane deformability) [23,38].

Primary and secondary prevention of vascular diseases is currently the basic weapon in the fight against stroke and heart attack [28,39,40]. Among the drugs used, an important role is played by preparations lowering the level of lipids in the blood, including statins. Analysis of the values of hemorheological and biochemical parameters conducted in the study showed that the value of the Quemada model parameter k_0_, describing the tendency of erythrocytes to aggregate and the parameter k_∞_ expressing the susceptibility of erythrocytes to deformation in the group of patients not taking statins, has lower values compared to the control group (Table 1). In the group of patients with silent ischemic foci of the brain not taking statins, the IgM concentration and the albumin/globulin index value are lower, but the ESR value is higher (Table 2). The lower value of IgM and albumin globulin ratio in this group of patients corresponds with literature data [41,42,43] as a marker of an increased risk of vascular disorders, including ischemic foci of the brain. A low value of the albumin/globulin ratio indicates an increased content of globulins in the blood serum in the group of patients not undergoing statin therapy, and a reduced value of IgM may indicate a higher content of fibrinogen in the plasma, contributing to increased plasma viscosity [4,5]. A higher ESR value in this group of patients may be related to a higher plasma viscosity ηp caused by a higher concentration of globulins resulting from a reduced value of the albumin/globulin ratio [4,44,45].

Analyzing the data obtained in this study, it can be stated that in the group of patients with clinically silent ischemic foci of the brain who were not treated with statins, compared to the control group also not subjected to statin therapy, red blood cells demonstrate better deformability and form shorter rolls, which may be an expression of the occurring autoregulation processes associated with the increase in plasma viscosity ηp [6,7,46,47] in order to maintain blood flow in the microcirculation in the conditions of the formation of ischemic foci of the brain.

Based on the analysis of the results obtained in the study for the group of patients with ischemic brain lesions compared to the control group undergoing statin therapy (Table 3), it was found that in the group of patients there was a reduced plasma viscosity ηp, as well as a lower concentration of IgM and cholesterol and the albumin/globulin index (Table 4). The reduced value of plasma viscosity in the group of patients with ischemic brain lesions may result from the dependence of plasma viscosity on factors such as the type and duration of the disease, comorbidities, and medications taken [3,4,11,15,17,18,35]. The change in the value of plasma viscosity may also result from the development of mechanisms regulating hemorheological properties. This mechanism is not yet fully understood [5,7,9,17,48,49].

The decreased IgM concentration observed in the group of patients treated with statins and the lower albumin/globulin ratio (Table 4) in comparison to the control group treated with statins are similar to those observed in the comparison of the group of patients and the control group not taking statins (Table 2). On the other hand, the decreased cholesterol concentration observed in the group of patients taking statins in comparison to the control group also taking statins (Table 4) may be related to the implementation of other hypolipidemic procedures, such as a more restrictive diet [50,51,52,53]. Lower total serum cholesterol concentration, mainly LDL fraction, results in lower plasma viscosity [22,54]. The lower plasma viscosity value observed in the study correlates with lower serum cholesterol concentration in the group of patients with clinically silent ischemic foci of the brain treated with statins compared to untreated patients. These results are confirmed in the literature [50,55].

The ability of erythrocytes to deform results from external viscosity related to the mechanical properties of the erythrocyte cell membrane, its internal viscosity resulting from the concentration of hemoglobin, pH, and osmolarity of blood, and disorders in electrolyte transport [38]. Moreover, the deformability of red blood cells depends not only on mechanical properties but is also related to their metabolism [21,24,56]. The results obtained in the study, in the group of patients with clinically silent ischemic foci of the brain with implemented statin therapy, indicate increased stiffening of erythrocytes in this group of patients with a simultaneous decrease in cholesterol values (Table 5 and Table 6). High cholesterol content in the plasma worsens the elasticity of erythrocytes [50], but the implementation of statin therapy improves the cholesterol/phospholipids ratio of the erythrocyte cell membrane and causes increased deformability in the group of patients with familial hypercholesterolemia [57,58,59]. The cholesterol/phospholipids index value reflects the cholesterol content in the red blood cell membrane. Increased cholesterol content in the membrane causes erythrocyte stiffening [22,50]. The results presented in this paper indicate that statin therapy used in some patients with clinically silent ischemic foci of the brain did not improve erythrocyte elasticity. The differences in the results obtained in this paper in relation to the literature data [21,58,59] may result from coexisting diseases that modulate the rheological properties of blood in the deformability of erythrocytes. The deformability of red blood cells is the end result of the action of many factors, both structural and metabolic [60,61], which were not analyzed in this paper.

**Figure 1:**
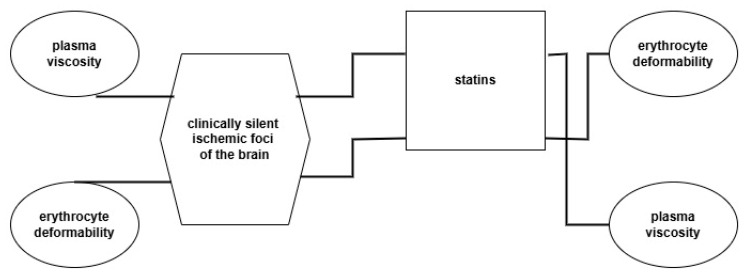
The scheme of changes in hemorheological parameters affected by the studied disorders and their reaction to statins.

It is well established in the literature that statins, beyond lowering serum cholesterol, can also alter membrane cholesterol composition, thereby affecting the integrity and function of lipid rafts. These microdomains play a key role in modulating cellular signaling, inflammation, and even viral entry. In erythrocytes, changes in lipid raft structure may influence membrane fluidity and deformability, which are relevant to hemorheological properties [62,63]. These analyses indicate the great importance of these processes and require further in-depth analysis in future studies (Figure 1).

## 4. Materials and Methods

The study was conducted in a total group of 69 patients, including 45 women and 24 men aged 25 to 83, hospitalized at the Neurology Department of the Municipal Hospital in Poznań for diagnostics due to ailments related to the nervous system and risk factors for cerebrovascular diseases. Written consent was obtained from patients in accordance with the decision of the bioethics committee No. 1012-1009. When including patients in the study, the results of neuroimaging studies, i.e., magnetic resonance imaging and 64-row computed tomography were taken into account, which confirmed the presence of patterns of changes in these patients that were characteristic of focal ischemic brain lesions. Computed tomography of the head was performed in 24 patients; the remaining part, i.e., 45 patients, underwent magnetic resonance imaging of the head. The changes found in the brain in the abovementioned studies met the radiological criteria for ischemic changes [64]. All patients routinely underwent blood count, sugar, urea, serum creatine levels, thyroid tests, liver function tests, lipid profiles, fibrinogen tests, general urine tests, and chest X-rays. The control group consisted of patients diagnosed in the department due to nervous system complaints; however, the condition for including a patient in the control group was a neurological examination in which no deficits were found and the presence of ischemic brain foci was excluded in neuroimaging studies performed in these patients. The control group included 17 people aged 25 to 77. All patients had their neurological condition checked several times. Similarly to the patients in the study group, the control group was burdened with risk factors for cerebrovascular diseases. The exclusion criterion for the study was a history of ischemic stroke and transient ischemic attack. The patients in the study group had the following concomitant diseases: hypertension (29 people), pre-cranial artery atherosclerosis (23 people), ischemic heart disease (14 people), and diabetes (9 people). In the control group, 6 people suffered from hypertension, 1 from diabetes, and 2 people had confirmed pre-cerebral artery atherosclerosis.

In order to check whether statin therapy affects hemorheological properties, two subgroups were distinguished among patients as follows: patients taking statins (*n* = 19) (zocor-simvastatin 10 or atorvasterol 20)—average age of 64 years; and those not taking statins (*n* = 50)—average age of 62 years. In the control group only 3 persons took statins. The average age in the control group not taking statins (*n* = 14) was 57 years.

Hemorheological tests were performed using the Contraves LS40 oscillation–rotation rheometer. Whole blood viscosity was measured at a decreasing shear rate γ’ in the range from 100 s^−1^ to 0.01 s^−1^ over 5 min. Plasma viscosity was measured using the linear regression method based on measurements and using the equilibrium curve in the range from 30 to 100 s^−1^. The hematocrit value was determined for each sample using the centrifugation method. The period between blood collection and the examination did not exceed four hours. The analysis of the erythrocyte aggregation and deformation tendency was performed by the indirect method using the Quemada rheological model [17,65].

## 5. Conclusions

In the group of patients treated with statins, a decrease in plasma viscosity and deterioration of erythrocyte deformability were demonstrated, which may suggest a dual effect of statins on hemorheological properties in patients with clinically silent foci of cerebral ischemia.

## Figures and Tables

**Table 1:** Values of hemorheological parameters in the control group and in the group of patients with clinically silent ischemic foci of the brain not taking statins.

Rheological Parameters	Control Group (Without Statins) *n* = 14	*p*	Patient Group (Without Statins) *n* = 50
Hematocrit [%]	41.3 ± 0.9	0.44	42.0 ± 0.4
Plasma viscosity η_p_ [mPas]	1.42 ± 0.04	0.73	1.41 ± 0.01
Relative viscosity of whole blood at a shear rate of 0.1 [s^−1^]	28 ± 4	0.49	26 ± 1
Relative viscosity of whole blood at a shear rate of 1 [s^−1^]	14 ± 1	0.64	13.4 ± 0.6
Relative viscosity of whole blood at a shear rate of 10 [s^−1^]	5.7 ± 0.2	1	5.7 ± 0.1
Relative viscosity of whole blood at a shear rate of 100 [s^−1^]	3.32 ± 0.08	0.44	3.43 ± 0.07
Queamada model parameter k_0_	4.33 ± 0.04	0.005	4.17 ± 0.03
Queamada model parameter k_∞_	1.79 ± 0.02	0.05	1.69 ± 0.03
Queamada model parameter γ’_c_	6.2 ± 0.9	0.35	5.6 ± 0.3

**Table 2:** Biochemical parameter values in the control group and in the group of patients with clinically silent ischemic foci of the brain not taking statins.

Parameters Biochemical	Control Group (Without Statins) *n* = 14	*p*	Patient Group (Without Statins) *n* = 50
fibrinogen	4.1 ± 0.3	0.34	3.7 ± 0.2
IgM	1.4 ± 0.1	0.004	1.01 ± 0.07
IgG	9.4 ± 0.5	0.11	10.7 ± 0.4
IgA	2 ± 1	0.6	2.3 ± 0.1
ESR	8.4 ± 1.1	0.049	12 ± 1
Albumins/globulins	1.83 ± 0.12	0.009	1.56 ± 0.04
Total protein	70.3 ± 1.5	0.39	71.8 ± 0.8
Glucose	5.08 ± 0.09	0.22	5.46 ± 0.15
Cholesterol	5.7 ± 0.3	0.47	5.45 ± 0.16

**Table 3:** Values of hemorheological parameters in the control group and in the group of patients with clinically silent ischemic foci of the brain taking statins.

Rheological Parameters	Control Group (Statins) *n* = 3	*p*	Patient Group (Statins) *n* = 19
Hematocrit [%]	39.5 ± 0.9	0.21	41.6 ± 0.6
Plasma viscosity η_p_ [mPas]	1.54 ± 0.05	0.03	1.34 ± 0.03
Relative viscosity of whole blood at a shear rate of 0.1 [s^−1^]	22 ± 7	0.42	29 ± 3
Relative viscosity of whole blood at a shear rate of 1 [s^−1^]	11 ± 1	0.27	14 ± 1
Relative viscosity of whole blood at a shear rate of 10 [s^−1^]	5.7 ± 0.2	0.64	6.2 ± 0.4
Relative viscosity of whole blood at a shear rate of 100 [s^−1^]	3.32 ± 0.08	0.48	3.7 ± 0.2
Queamada model parameter k_0_	4.33 ± 0.14	0.72	4.27 ± 0.06
Queamada model parameter k_∞_	1.75 ± 0.05	0.56	1.86 ± 0.07
Queamada model parameter γ’_c_	6.2 ± 0.9	0.39	5.0 ± 0.5

**Table 4:** Biochemical parameter values in the control group and in the group of patients with clinically silent ischemic foci of the brain taking statins.

Parameters Biochemical	Control Group (Statins) *n* = 3	*p*	Patient Group (Statins) *n* = 19
Fibrinogen	4.8 ± 0.8	0.07	3.6 ± 0.2
IgM	1.4 ± 0.1	0.05	0.95 ± 0.08
IgG	9.4 ± 0.5	0.88	9.7 ± 0.5
IgA	2 ± 1	0.65	2.4 ± 0.2
ESR	8.4 ± 1.1	0.76	11 ± 2
Albumins/globulins	1.83 ± 0.12	0.047	1.58 ± 0.04
Total protein	70.3 ± 1.5	0.9	71 ± 1
Glucose	5.08 ± 0.09	0.6	5.7 ± 0.3
Cholesterol	5.7 ± 0.3	0.04	4.5 ± 0.2

**Table 5:** Comparison of hemorheological values in the group of patients divided into those taking and not taking statins.

Rheological Parameters	Patient Group (Statins) *n* = 19	*p*	Patient Group (Without Statins) *n* = 50
Hematocrit [%]	41.6 ± 0.6	0.6	42.0 ± 0.4
Plasma viscosity η_p_ [mPas]	1.34 ± 0.03	0.007	1.41 ± 0.01
Relative viscosity of whole blood at a shear rate of 0.1 [s^−1^]	29 ± 3	0.23	26 ± 1
Relative viscosity of whole blood at a shear rate of 1 [s^−1^]	14 ± 1	0.61	13.4 ± 0.6
Relative viscosity of whole blood at a shear rate of 10 [s^−1^]	6.2 ± 0.4	0.1	5.7 ± 0.1
Relative viscosity of whole blood at a shear rate of 100 [s^−1^]	3.7 ± 0.2	0.11	3.43 ± 0.07
Queamada model parameter k_0_	4.27 ± 0.06	0.1	4.17 ± 0.03
Queamada model parameter k_∞_	1.86 ± 0.07	0.01	1.69 ± 0.03
Queamada model parameter γ’_c_	5.0 ± 0.5	0.3	5.6 ± 0.3

**Table 6:** Comparison of biochemical values in the group of patients divided into those taking and not taking statins.

Biochemical Parameters	Patient Group (Statins) *n* = 19	*p*	Patient Group (Without Statins) *n* = 50
fibrinogen	3.6 ± 0.2	0.l78	3.7 ± 0.2
IgM	0.95 ± 0.08	0.63	1.01 ± 0.07
IgG	9.7 ± 0.5	0.17	10.7 ± 0.4
IgA	2.4 ± 0.2	0.63	2.3 ± 0.1
ESR	11 ± 2	0.63	12 ± 1
Albumins/globulins	1.58 ± 0.04	0.77	1.56 ± 0.04
Total proteins	71 ± 1	0.58	71.8 ± 0.8
Glucose	5.7 ± 0.3	0.44	5.46 ± 0.15
Cholesterol	4.5 ± 0.2	0.002	5.45 ± 0.16

## Data Availability

The data presented in this study are available from the corresponding author upon reasonable request.
